# Disparities in Limb-Saving Surgery Persist Among Black Patients With Peripheral Artery Disease

**DOI:** 10.7759/cureus.105585

**Published:** 2026-03-21

**Authors:** William E Allen, Joshua D Greendyk, Syed F Haider, Afif Hossain, Abhishek Sharma

**Affiliations:** 1 Department of Medicine, Section of General Internal Medicine, Boston Medical Center, Boston, USA; 2 Department of Medicine, Division of Cardiology, Rutgers New Jersey Medical School, Newark, USA; 3 Department of Surgery, Yale School of Medicine, New Haven, USA

**Keywords:** amputation, atherosclerosis, chronic limb-threatening ischemia, endovascular, peripheral arterial disease

## Abstract

Background

Peripheral artery disease (PAD) affects many individuals in the United States and imposes substantial healthcare costs. Chronic limb-threatening ischemia (CLTI) is a severe form of PAD. This study evaluates racial disparities in access to different treatment approaches for CLTI, hypothesizing that patients of Black self-reported race may experience significant barriers to receiving open surgical bypass.

Methods

In this retrospective study, data were analyzed from the American College of Surgeons-National Surgical Quality Improvement Program (ACS-NSQIP) database. All recorded cases of surgical bypass, endovascular revascularization, or amputation below the knee for the treatment of CLTI from 2012 to 2021 were included in this analysis. Propensity score matching and univariate analyses were conducted to evaluate the association between self-identified race, treatment access, and clinical outcomes.

Results

A total of 42,889 cases of CLTI were identified. Black patients were less likely to undergo open surgery (5,000 (38.4%) vs. 13,981 (47.7%); p < 0.001) and more likely to undergo endovascular therapy (6,402 (49.2%) vs. 13,299 (45.3%); p < 0.001) and amputation (1,621 (12.4%) vs. 2,060 (7.0%); p < 0.001). Black patients also experienced higher rates of postoperative complications, including cardiac arrest, deep vein thrombosis, sepsis, myocardial infarction, ventilator dependence >48 hours, reoperation, 30-day mortality, and 30-day readmission. After propensity score matching and logistic regression, Black self-reported race remained an independent predictor of endovascular revascularization and amputation rather than open bypass.

Conclusions

This study revealed a significant racial disparity in access to open bypass among self-identified Black CLTI patients. Patients of Black self-reported race were more likely to experience postoperative complications or reoperation within 30 days following the operation. Although an endovascular technique may reduce 30-day complications, recent literature indicates that open bypass may improve long-term postoperative outcomes. These results highlight the need for further investigation to identify and mitigate barriers contributing to disparities.

## Introduction

Over eight million individuals in the United States have peripheral artery disease (PAD), with a cumulative management cost exceeding $21 billion annually [1]. In its most severe form, PAD can progress to chronic limb-threatening ischemia (CLTI), which has a two-year mortality rate as high as 40% and an estimated annual cost of $12 billion [2,3].

PAD is a chronic disease commonly resulting from atherosclerotic plaques that narrow the arterial lumen and impede blood flow to the distal extremities. Risk factors for the development and exacerbation of PAD include concurrent diagnoses of hypertension, hyperlipidemia, diabetes, and prior tobacco use [4]. An advanced complication of PAD, CLTI, is defined as ischemic pain at rest that persists for two or more weeks or is diagnosed by the presence of nonhealing wounds attributable to arterial occlusive disease [5]. Historically, the prevalence of PAD has varied by race and is highest in Black populations [6]. This phenomenon is often attributed to social determinants of health that create barriers to the receipt of adequate healthcare [7]. It is well established that social determinants of health, particularly those affecting Black and Hispanic populations, significantly influence cardiovascular outcomes, care delivery, and procedural utilization [8]. These disparities are not attributable to biological differences alone but are largely driven by structural and systemic factors that shape access to care, quality of treatment, and longitudinal disease management.

Management of CLTI includes local wound care to promote healing and prevent soft tissue infection, guideline-directed medical therapy to reduce atherosclerotic cardiovascular risk (ASCVD), and revascularization to restore perfusion [9]. Without timely revascularization, the risk of amputation approaches 25% within one year of diagnosis [10].

Currently, open surgical bypass and endovascular therapy represent the primary revascularization strategies for the treatment of CLTI [9]. Selection of the initial approach varies widely among providers and depends on several patient-specific factors, including vascular anatomy, pattern of arterial disease, operative risk, and the availability of suitable autogenous vein conduit. Patient preference, as well as physician training, technical expertise, and potential treatment bias, may also influence decision-making [11-13]. Existing literature remains mixed regarding the optimal revascularization strategy [14-18]. Although the recent BEST-CLI trial demonstrated superior outcomes with open surgical bypass compared with endovascular therapy, ongoing advancements in endovascular techniques may offer improved outcomes for selected patients [9]. The impact of variability in treatment selection on clinical outcomes in patients with CLTI remains incompletely understood [11,13,19].

Minimally invasive techniques have significantly advanced surgical care in recent years. However, not all groups of patients benefit from these medical advances equally. Patient race, ethnicity, gender, and socioeconomic status are potential factors influencing inequities in care. Prior evaluation of disparities in access to basic minimally invasive procedures is often limited to non-profit, academic medical centers [20].

Given the prevalence of CLTI, the significant risk of mortality, and the demonstrated advantages of surgical treatment, investigating treatment modalities in vulnerable patient populations is imperative. This study evaluates the association between race and the type of revascularization performed, using a multi-institutional database comprising governmental, municipal, academic, and community hospitals. We hypothesized that patients with self-reported Black race would be less likely to undergo open surgical bypass compared with other revascularization approaches.

This article was previously presented as a meeting abstract at the 2023 American Heart Association Annual Scientific Meeting on November 11, 2023.

## Materials and methods

Data acquisition

All demographic, perioperative, operative, and clinical variables were collected from the American College of Surgeons-National Surgical Quality Improvement Program (ACS-NSQIP) database for the years 2012-2021 [21]. The ACS-NSQIP is a nationally validated, outcomes-based, risk-adjusted, peer-controlled program for measuring and enhancing the quality of surgical care. All data were deidentified, and the study was conducted under an IRB exemption. The ACS-NSQIP and participating hospitals have not verified and are not responsible for the statistical validity of the data analysis or the conclusions drawn by the authors.

Patients who underwent surgical bypass, endovascular revascularization, or lower extremity amputation for the treatment of CLTI between 2012 and 2021 were identified and included in the study using Current Procedural Terminology (CPT) codes, stratified by open surgery, endovascular therapy, and amputation (Table 1). Non-CLTI amputations were excluded by integrating procedure codes with diagnostic criteria, restricting the cohort to encounters with concurrent ICD diagnosis codes consistent with CLTI (e.g., rest pain, ulceration, or gangrene). Amputations associated with alternative primary indications, including trauma and malignancy, were also excluded.

**Table 1:** CPT procedure codes used in this study CPT, Current Procedural Terminology

Surgery type	CPT codes
Open surgery	35537, 35538, 35539, 35540, 35556, 35558, 35563, 35565, 35566, 35570, 35571, 35583, 35585, 35587, 35621, 35623, 35637, 35638, 35646, 35647, 35654, 35656, 35661, 35663, 35665, 35666, 35671
Endovascular therapy	37221, 37222, 37223, 37224, 37225, 37226, 37227, 37228, 37229, 37230, 37231, 37232, 37233, 37234, 37235
Amputation	27290, 27295, 27590, 27591, 27596, 27598, 27881, 27888, 27889, 27882, 27886, 25900, 25905, 25920, 25927, 26910, 26951, 26952

Variables of interest

Demographic variables assessed included patient self-identified race (White or Black), age greater than 60 years, and sex. Relevant preoperative comorbidities included American Society of Anesthesiologists (ASA) class >2, current or past smoking within one year, type 2 diabetes, congestive heart failure, hypertension managed with at least one medication, kidney failure (blood urea nitrogen or creatinine >3 mg/dL within 24 hours), chronic steroid use, current bleeding disorder, and chronic obstructive pulmonary disease (COPD). Postoperative complications were categorized as any, minor, or severe. Any complication included superficial incisional surgical site infection (SSI), deep incisional SSI, organ-space SSI, wound disruption, pneumonia, unplanned intubation, pulmonary embolism (PE), failure to wean from the ventilator within 48 hours, progressive renal insufficiency, acute renal failure (ARF), UTI, ischemic stroke or cerebrovascular accident (CVA), cardiac arrest, myocardial infarction (MI), deep vein thrombosis (DVT), return to the operating room, and sepsis. Minor complications included UTI, superficial SSI, pneumonia, and unplanned readmission, while severe complications included sepsis, septic shock, MI, cardiac arrest, deep wound infection, deep SSI, organ/space SSI, wound dehiscence, PE, DVT, progressive renal insufficiency, ARF, CVA, need for transfusion, unplanned reintubation, failure to wean from the ventilator within 48 hours, unplanned readmission, or unplanned reoperation. Complications were compiled into the any, minor, and severe groups as previously described [21].

Primary and secondary outcomes

The primary outcome of interest was an open surgical approach to evaluate differences in procedural utilization, while secondary analyses assessed major postoperative complications. Demographic characteristics and comorbidities of patients by self-identified race were compared using univariate and multivariate analyses. Chi-squared tests and logistic regression were performed on all categorical variables, with p-values <0.05 considered statistically significant. All variables were subsequently used in a 1:1 propensity score-matched analysis between Black and White patients.

Propensity score matching

To isolate race as an independent predictor of postoperative outcomes while controlling for other clinical variables, demographic characteristics, and comorbidities stratified by self-identified race were summarized and compared. These variables were used in a subsequent propensity score-matched analysis between Black and White patients. Matching was performed using a nearest-neighbor algorithm with a 1:1 ratio to reduce baseline differences while balancing all available clinical variables except race. Caliper width was set at 0.2 standard deviations of the propensity score. Matching balance was evaluated using standardized mean differences, with thresholds <0.1 indicating adequate covariate balance.

Mediation analysis

To evaluate whether the operative approach mediates the association between race and postoperative complications, a mediation analysis was conducted using Black race as the independent variable, open surgical approach as the mediator, and postoperative complications as the dependent variable, following previously described methodology [22]. A three-regression framework was applied, first regressing race on postoperative complications to estimate the total effect, then regressing race on open surgical approach to assess the association between race and the proposed mediator, and finally regressing race and operative approach simultaneously on postoperative complications to estimate the direct effect of race while controlling for surgical approach. Mediation was supported if all three models were statistically significant and the OR for race in the final model was attenuated relative to the total effect. The magnitude of mediation was quantified using the mediation ratio, calculated as follows:

$\text{Mediation ratio} = 1 - \left( \frac{\text{Direct effect}}{\text{Total effect}} \right),$

which, multiplied by 100%, yielded the proportion of the total effect of race on postoperative complications attributable to operative approach [21]. This calculation was performed for all statistically significant mediated associations.

Statistical analysis

Rates of procedural utilization were computed and compared across clinical and demographic categories. Pearson’s χ² and independent-samples t-tests were used to analyze categorical and continuous variables, respectively. Variables significantly associated with race on univariate analysis (p ≤ 0.05) were entered into binary logistic regressions to identify independent predictors of race on demographics and clinical outcomes. Model fit was assessed using a likelihood ratio test comparing the final model with the intercept-only model, and model explanatory power was summarized using pseudo R² statistics. p-Values ≤0.05 were considered statistically significant, and all tests were two-sided. Analyses were conducted using IBM SPSS Statistics for macOS, Version 25.0 (Released 2017; IBM Corp., Armonk, NY, USA).

## Results

Patient clinical demographics

The initial query of the ACS-NSQIP database returned 50,306 cases of treated CLTI. After excluding 7,417 cases with unknown race, the final study cohort included 42,889 patients. The median patient age was 69 years (IQR 61-78), and 40.2% were female. Most individuals reported being White (n = 29,691; 69.2%). Univariate analysis indicated that White patients were more likely to undergo open surgery, be male, have a history of smoking, possess a bleeding disorder, use steroids for chronic disease management, and suffer from COPD. In contrast, Black patients were more likely to undergo endovascular therapy (49.2% vs. 45.3%; p < 0.001) or amputation (12.4% vs. 7.0%; p < 0.001) and were more likely to have diabetes, congestive heart failure, medicated hypertension, kidney failure, and a history of transfusion (Table 2).

**Table 2:** Univariate analysis and multivariate logistic regression of self-identified race with surgical approach, demographics, and preoperative comorbidities Data are presented as n (%). p-Values <0.05 were considered statistically significant using Pearson’s χ² test. Some variables may not sum to the total number of cases due to missing data, although such instances were few. ASA, American Society of Anesthesiologists physical status classification system; CHF, congestive heart failure; COPD, chronic obstructive pulmonary disease

Variables	White patients, N = 29,691, n (%)	Black patients, N = 13,198, n (%)	Test value (χ²)	p-Value
Open surgery	13,981 (47.7%)	5,000 (38.4%)	313.72	<0.001
Endovascular	13,299 (45.3%)	6,402 (49.2%)	50.8	<0.001
Amputation	2,060 (7.0%)	1,621 (12.4%)	332.57	<0.001
Age >60 years	23,251 (78.3%)	9,525 (72.2%)	155.31	<0.001
Male	18,219 (62.0%)	7,038 (53.7%)	293.54	<0.001
Female	11,174 (38.0%)	6,058 (46.3%)	259.77	<0.001
ASA >2	27,787 (95.8%)	12,447 (95.9%)	8.21	0.294
Smoker	12,336 (41.5%)	4,665 (35.3%)	146.87	<0.001
Type 2 diabetes	12,576 (42.4%)	7,315 (55.4%)	627.48	<0.001
CHF	1,428 (4.8%)	728 (5.5%)	9.55	0.003
Hypertension	24,334 (82.0%)	11,522 (87.3%)	190.3	<0.001
Kidney failure	1,804 (6.2%)	2,357 (17.9%)	1,447.96	<0.001
Steroid use	1,863 (6.3%)	743 (5.6%)	6.66	0.005
Bleeding disorder	8,833 (29.7%)	3,557 (27.0%)	34.84	<0.001
COPD	5,155 (17.4%)	1,104 (8.4%)	593.41	<0.001
Transfusion	571 (1.9%)	486 (3.7%)	117.64	<0.001

Association of race/ethnicity with complication rates

Table 3 summarizes operative course and surgical outcomes. Black patients had significantly longer postoperative length of stay and greater total length of stay compared with White patients. They also experienced higher rates of several postoperative complications, including cardiac arrest, DVT, systemic sepsis, septic shock, MI, ventilator dependence >48 hours, reoperation, 30-day mortality, and 30-day readmission. Overall complication rates, including minor and severe complications, were higher among Black patients. In contrast, White patients had longer operative times and higher rates of superficial SSI. No significant differences were observed in organ space SSI, wound dehiscence, pneumonia, CVA, ARF, or UTI.

**Table 3:** Univariate analysis of the association of self-reported race with operative course and surgical complications ᵃ Any complication ᵇ Minor complication ᶜ Severe complication Data are presented as n (%) or mean ± SD. p-Values were considered significant at <0.05 using ^*^independent samples Student’s t-test or Pearson’s χ². ARF, acute renal failure; DVT, deep vein thrombosis; LOS, length of stay; MI, myocardial infarction; PE, pulmonary embolism; SSI, surgical site infection

Variables	White patients, N = 29,691, n (%)	Black patients, N = 13,198, n (%)	Test statistics	p-Value
Operative time (minutes)	175 ± 113	158 ± 114^*^	14.3	<0.001
Postoperative LOS (days)	4.40 ± 7.7	5.10 ± 9.8^*^	7.27	<0.001
Total LOS (days)	5.95 ± 9.4	7.59 ± 11.8^*^	14.16	<0.001
Superficial SSIᵃ^,^ᵇ	1,230 (4.1%)	329 (2.5%)	71.00	<0.001
Deep SSIᵃ^,^ᶜ	464 (1.6%)	177 (1.3%)	3.05	0.044
Organ space SSIᵃ^,^ᶜ	186 (0.6%)	90 (0.7%)	0.44	0.275
Dehiscenceᵃ^,^ᶜ	333 (1.1%)	153 (1.2%)	0.12	0.386
Pneumoniaᵃ^,^ᵇ^,^ᶜ	672 (2.3%)	301 (2.3%)	0.01	0.470
Reintubationᵃ^,^ᶜ	599 (2.0%)	317 (2.4%)	6.46	0.006
PEᵃ^,^ᶜ	45 (0.2%)	32 (0.2%)	4.21	0.027
UTIᵃ^,^ᵇ	430 (1.4%)	200 (1.5%)	0.28	0.312
Cardiac arrestᵃ^,^ᶜ	326 (1.1%)	210 (1.6%)	18.01	<0.001
DVTᵃ^,^ᶜ	190 (0.6%)	111 (0.8%)	5.32	0.012
Systemic sepsisᵃ^,^ᶜ	641 (2.2%)	419 (3.2%)	39.11	<0.001
Septic shockᵃ^,^ᶜ	303 (1.0%)	204 (1.5%)	21.57	<0.001
Cerebrovascular attackᵃ^,^ᶜ	182 (0.6%)	82 (0.6%)	0.01	0.486
MIᵃ^,^ᶜ	748 (2.5%)	245 (1.9%)	17.75	<0.001
ARFᵃ^,^ᶜ	170 (0.6%)	88 (0.7%)	1.36	0.136
Ventilator >48 hoursᵃ^,^ᶜ	379 (1.3%)	203 (1.5%)	4.67	0.017
Any complication	6,770 (22.8%)	3,152 (23.9%)	6.00	0.007
Minor complication	5,646 (19.0%)	2,667 (20.2%)	8.30	0.002
Severe complication	3,836 (12.9%)	1,977 (15.0%)	33.09	<0.001
30-day mortalityᵃ^,^ᶜ	712 (2.4%)	514 (3.9%)	27.21	<0.001
Reoperationᵃ^,^ᶜ	3,862 (13.0%)	1,933 (14.6%)	21.00	<0.001
30-day readmissionᵃ^,^ᵇ^,^ᶜ	4,434 (14.9%)	2,240 (17.0%)	28.89	<0.001

Table 4 presents the results of regression analyses evaluating operative course and surgical outcomes. Compared with White patients, Black patients had higher odds of PE, systemic sepsis, and septic shock. Conversely, Black patients had lower odds of superficial SSI, deep SSI, and MI.

**Table 4:** Binary logistic regression analysis of self-reported race on postoperative outcomes ^*^ Adjusted for age, gender, smoking status, congestive heart failure, diabetes, hypertension, chronic obstructive pulmonary disease, bleeding disorder, history of transfusion, and kidney failure. 95% CIs are significant if the range does not include 1. ARF, acute renal failure; DVT, deep vein thrombosis; MI, myocardial infarction; PE, pulmonary embolism; SSI, surgical site infection

Variables	Adjusted OR^*^	95% CI	p-Value
Superficial SSI	0.552	0.477-0.639	<0.001
Deep SSI	0.755	0.618-0.922	0.006
Reintubation	1.095	0.916-1.309	0.319
PE	1.806	1.106-2.950	0.018
Cardiac arrest	1.094	0.883-1.356	0.413
DVT	1.227	0.958-1.570	0.105
ARF	1.175	0.941-3.193	0.256
Sepsis	1.395	1.195-1.628	<0.001
Septic shock	1.422	1.154-1.752	0.001
MI	0.586	0.494-0.696	<0.001
Ventilator >48 hours	0.718	0.453-1.138	0.159
Any complication	1.066	0.947-1.198	0.290
Minor complication	0.99	0.853-1.148	0.891
Severe complication	0.903	0.804-1.014	0.085
30-day mortality	1.103	0.982-1.341	0.062
Reoperation	1.017	0.912-1.133	0.067
30-day readmission	1.127	0.975-1.303	0.105

Propensity score matching and mediation analysis

A 1:1 propensity score matching algorithm was applied to the CLTI cohort (Table 5), yielding 9,552 patients identifying as White and 9,499 identifying as Black. The slight difference in group sizes reflects cases that could not be matched within the designated caliper or common support region and were therefore excluded from the matched sample. Subsequent logistic regression analysis of race and surgical approach demonstrated that, within the CLTI cohort, Black self-reported race independently predicted receipt of endovascular therapy and a higher likelihood of amputation. Logistic regression of postoperative complications indicated that, among CLTI patients, non-White patients were more likely to experience any complications, minor complications, severe complications, unplanned reoperation, and unplanned readmission (Table 5).

**Table 5:** Multivariate logistic regression of Black self-reported race on surgical approach and postoperative complications in propensity score-matched cohorts

Variables	OR	95% CI	p-Value
Endovascular recanalization	1.049	1.002-1.099	0.040
Amputation	2.029	1.887-2.182	<0.001
Any complications	1.071	1.015-1.130	0.013
Minor complications	1.069	1.010-1.132	0.021
Severe complications	1.220	1.144-1.302	<0.001
Unplanned readmission	1.153	1.084-1.226	<0.001
Unplanned reoperation	1.188	1.113-1.267	<0.001

Because Black self-reported race was associated with both postoperative complications and receipt of endovascular revascularization or amputation, a mediation analysis was performed. When postoperative complications were regressed on both Black self-reported race and surgical approach, race remained a significant predictor of any complication (OR 1.071, 95% CI 1.015-1.130, p = 0.013) (Table 5). The mediation analysis showed that the surgical approach accounted for 6.2% of the total effect of Black self-reported race on postoperative outcomes.

## Discussion

The goal of this study was to better understand the relationship between patient self-reported race, operative approach for the treatment of CLTI, and postoperative outcomes. Our findings reveal a significant racial disparity in access to open bypass surgery, which is clinically meaningful because disease characteristics directly inform revascularization strategy. Patients with more extensive, calcified, or multilevel arterial disease are more amenable to durable revascularization with open bypass and may derive greater long-term benefit from surgical intervention, whereas less complex lesions are often treated with endovascular approaches. Additionally, self-reported Black race was associated not only with increased postoperative complications but also with higher rates of unplanned readmission and unplanned reoperation. These findings highlight two key issues in CLTI management: underutilization of open bypass among Black patients and higher overall morbidity compared with non-Hispanic White patients. Together, these results underscore the need for further research to identify and address racial disparities and barriers to care in CLTI management.

Racial disparities across surgical subspecialties have been extensively documented, with minority patients often experiencing higher crude mortality rates, longer lengths of stay, and increased treatment costs [22,23]. More recently, disparities in access to common procedures such as total knee arthroplasty, total hip arthroplasty, and heart valve replacement have persisted despite nationwide initiatives to reduce gaps between White and Black patients [24]. These disparities have also been observed in settings with relatively uniform economic status and insurance coverage, such as within the Veterans Health Administration and Medicare programs [25]. Consistent with existing literature, our analysis shows that Black patients are significantly less likely to receive open bypass compared with endovascular therapy for CLTI. Although our database lacks detailed socioeconomic data, these findings are supported by the concept that endovascular procedures may cost healthcare payers less than open bypass or primary amputation due to shorter hospital stays and lower resource utilization [26].

An endovascular-first approach to infra-inguinal CLTI has been associated with reduced early morbidity compared with open bypass; however, recent evidence suggests that open bypass may provide superior long-term outcomes in patients with CLTI [9]. In a clinical trial involving 1,830 patients with CLTI and comorbid infra-inguinal PAD, those with an adequate great saphenous vein experienced significantly fewer major adverse limb events or deaths when receiving open bypass compared with endovascular revascularization over several years of follow-up [9]. Our findings highlight racial disparities in access to open bypass vs. endovascular therapy for patients with comorbid CLTI and infra-inguinal PAD, supporting the notion that implicit bias may influence clinicians’ awareness of worse outcomes in minority populations and limit earlier or more aggressive treatment for these patients [27].

The effects of physician bias and lower patient health literacy are also evident in the management of diabetes among Black populations, where prevalence rates could double by 2050 [28]. In the context of CLTI, prior studies have identified low socioeconomic status and Black race as independent risk factors for delayed presentation, higher amputation rates, and increased emergency-to-elective surgery ratios for advanced limb ischemia [29]. Even after accounting for differences in healthcare access, lower household income, and insurance status, patients with critical limb ischemia are more likely to undergo amputation and less likely to receive limb-salvaging revascularization [30]. These disparities likely reflect downstream factors not fully captured by these variables, including delayed presentation with more advanced disease, higher comorbidity burden (e.g., diabetes, chronic kidney disease, and infection), and differences in referral patterns to vascular specialists or high-volume centers. Hospital-level factors such as resource availability, surgical vs. endovascular expertise, and institutional practice patterns may also influence procedural selection. Structural factors, including implicit bias and differences in longitudinal care access, may further reduce utilization of limb-salvaging interventions. This issue is particularly important because mortality within 30 days of lower extremity amputation is nearly double that of patients who do not undergo amputation [31]. These findings emphasize the need for future research addressing structural disparities that may shape CLTI treatment strategies in minority populations.

Limitations

Several limitations should be noted. First, the ACS-NSQIP database lacks geographic, socioeconomic, and hospital identifiers, limiting detailed demographic stratification. The original CLTI cohort included nearly four times as many White patients as Black patients, resulting in the exclusion of many White patients for the 1:1 propensity matching. While this data loss may be problematic, the model allowed us to control for demographic and comorbid confounding variables that could otherwise obscure relationships between race, surgical approach, and postoperative outcomes.

Second, the ACS-NSQIP database provides only a 30-day postoperative window. Ideally, a longitudinal follow-up of one year or more would allow assessment of subsequent revascularization, amputation, or serious health complications after endovascular or open interventions. Third, including patients undergoing open bypass, endovascular intervention, and primary amputation within a single analytic cohort introduces heterogeneity in both disease severity and treatment intent, which may confound comparisons.

Fourth, the dataset lacks granular covariates such as socioeconomic factors, hospital-level characteristics, lesion anatomy, and conduit availability, which are known to influence revascularization strategy and outcomes, limiting risk adjustment. Finally, the exclusion of patients with unknown race may introduce selection bias and affect generalizability. Additionally, while ACS-NSQIP represents a national patient sample, our findings may not generalize to specific healthcare models, institutions, or other populations.

## Conclusions

This study provides insight into the relationship between race, surgical approach, and postoperative complications in the management of CLTI. Our analysis identified differences in treatment patterns, with patients of Black self-reported race having higher odds of receiving endovascular revascularization or amputation rather than open bypass. Black patients also experienced higher rates of postoperative complications and reoperation within 30 days. These findings highlight significant associations that warrant further investigation to better understand and address potential disparities in care delivery and outcomes.
